# Cerebral venous steal equation for intracranial segmental perfusion pressure predicts and quantifies reversible intracranial to extracranial flow diversion

**DOI:** 10.1038/s41598-021-85931-x

**Published:** 2021-04-08

**Authors:** Mindaugas Pranevicius, Henrikas Pranevicius, Osvaldas Pranevicius

**Affiliations:** 1 Albert Einstein College of Medicine, Bronx, NY USA; 2 Kaunas University of Technology, Simulation of Complex Systems, Kaunas, Lithuania; 3 Department of Anesthesiology, New York-Presbyterian/Queens, New York, NY USA; 4 Department of Anesthesiology, Jacobi Medical Center, 1400 Pelham Pk. S, Bronx, NY 10461 USA

**Keywords:** Blood flow, Brain injuries, Stroke

## Abstract

Cerebral perfusion is determined by segmental perfusion pressure for the intracranial compartment (SPP), which is lower than cerebral perfusion pressure (CPP) because of extracranial stenosis. We used the Thevenin model of Starling resistors to represent the intra-extra-cranial compartments, with outflow pressures ICP and Pe, to express SPP = Pd–ICP = FFR*CPP–Ge(1 − FFR)(ICP–Pe). Here Pd is intracranial inflow pressure in the circle of Willis, ICP—intracranial pressure; FFR = Pd/Pa is fractional flow reserve (Pd scaled to the systemic pressure Pa), Ge—relative extracranial conductance. The second term (cerebral venous steal) decreases SPP when FFR < 1 and ICP > Pe. We verified the SPP equation in a bench of fluid flow through the collapsible tubes. We estimated Pd, measuring pressure in the intra-extracranial collateral (supraorbital artery) in a volunteer. To manipulate extracranial outflow pressure Pe, we inflated the infraorbital cuff, which led to the Pd increase and directional Doppler flow signal reversal in the supraorbital artery. SPP equation accounts for the hemodynamic effect of inflow stenosis and intra-extracranial flow diversion, and is a more precise perfusion pressure target than CPP for the intracranial compartment. Manipulation of intra-extracranial pressure gradient ICP–Pe can augment intracranial inflow pressure (Pd) and reverse intra-extracranial steal.

## Introduction

Cerebral perfusion pressure is not the sole determining factor of cerebral perfusion in the presence of extracranial stenosis^1^. Intra and extracranial vascular supply has multiple anastomoses^2^, which allows inflow pressure to equilibrate between intra and extracranial compartments. However, when intracranial pressure is higher than extracranial, blood flow is diverted extracranially.

We extrapolated findings of our earlier cerebral venous steal model^3^ to assess the distribution of flow between the intra and extracranial compartments, and to quantify segmental perfusion pressure (SPP) for the intracranial compartment which would account for extracranial stenosis.

## Results

We derived the intracranial segmental perfusion pressure equation, verified it in a physical bench and healthy volunteer, then simulated cerebral blood flow autoregulation and perfusion pressure in low-flow states.

### Segmental perfusion pressure (SPP) equation for the intracranial compartment

**Figure 1:**
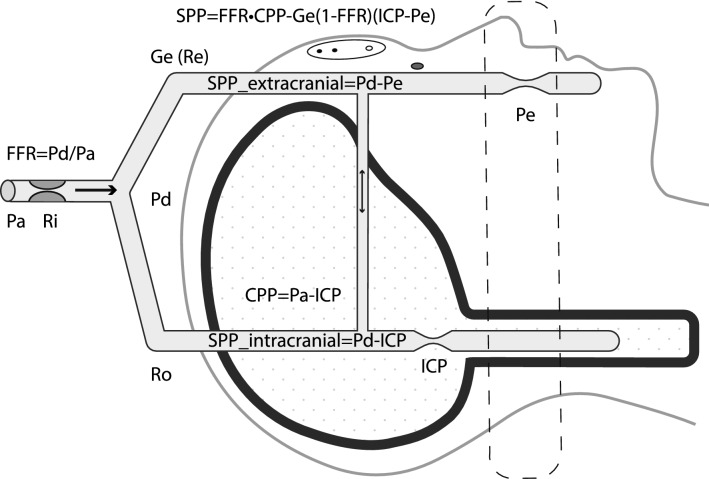
Schematic representation of intra-extracranial flow distribution. Despite the multitude of arterial and venous extra-intracranial supply pathways and anastomoses, all blood flow to the head is divided into the intracranial and extracranial, with corresponding compartmental pressures ICP and Pe. Extracranial compartment pressure is either atmospheric or venous (whichever is higher). Common inflow pressure Pd (equilibrated at the circle of Willis) is lower than systemic pressure Pa due to the common inflow resistance Ri—which determines fractional flow reserve- FFR. Pd can be estimated by measuring pressure in the intra-extracranial collateral—we measured supraorbital artery pressure using maximal photoplethysmographic oscillation criteria. Segmental perfusion pressure of the intracranial compartment (SPP = Pd–ICP), rather than cerebral perfusion pressure (CPP = Pa–ICP), is the driving gradient for the cerebral blood flow. SPP_intracranial was expressed using Starling resistor equations for the intra and extra-cranial compartments: SPP = FFR*CPP-Ge*(1 − FFR)*(ICP–Pe). If FFR is less than 1, and ICP > Pe, Pd is reduced not only by the inflow resistance (first term), but also by the additional pressure drop due to the flow diversion from the intracranial compartment (Ro) to the extracranial compartment (Re) with relative conductance Ge. The second term in the equation (intra-extracranial steal) reverses to the extra-intracranial augmentation when Pe > ICP. To investigate extra-intracranial flow distribution in a healthy volunteer, we manipulated extracranial outflow pressure Pe. Antegrade flow in the supraorbital artery (intra-extracranial steal) was reversed during stepwise inflation of the infraorbital cuff demonstrating the feasibility of intracranial blood flow augmentation.

Applying the Thevenin equivalent model to describe the distribution of the blood flow between intracranial and extracranial compartments of the head, we derived the segmental cerebral perfusion pressure equation for the intracranial compartment (Fig. 1, Appendix):

1 $${\text{SPP }} = {\text{ Pd}} - {\text{ICP }} = {\text{ FFR}} \cdot {\text{CPP }} - {\text{ Ge}} \cdot \, \left( {{1} - {\text{FFR}}} \right) \, \cdot \, \left( {{\text{ICP}} - {\text{Pe}}} \right)$$

In this equation, SPP is segmental perfusion pressure for the intracranial compartment, Pd—intracranial inflow pressure, ICP—intracranial pressure, Pa—systemic arterial pressure, FFR—Pd/Pa is the fractional flow reserve of the common inflow, CPP = Pa–ICP is cerebral perfusion pressure, Ge is the ratio of extracranial vascular conductance to total (intra-extracranial) vascular conductance, and Pe is extracranial outflow pressure.

### Bench with a collapsible tube to validate the SPP equation

**Figure 2:**
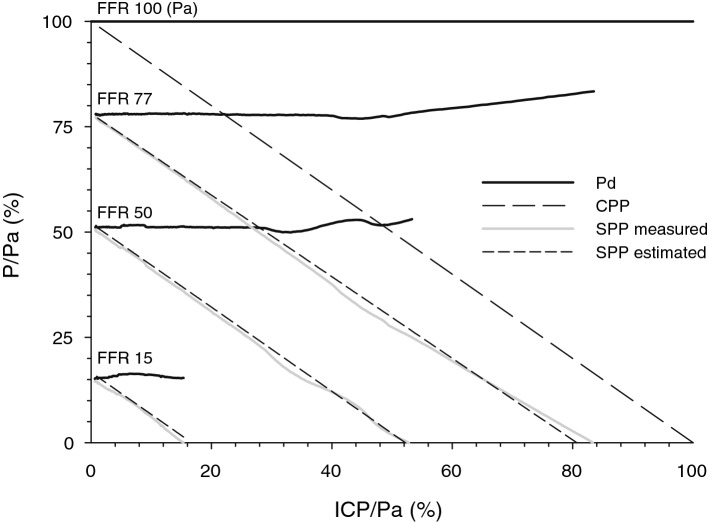
Bench verification of the segmental perfusion pressure (SPP) equation: intra-extracranial flow simulated by the irrigation set with inflow pressure Pa flowing through the adjustable clamp and dividing into two outflow pathways: collapsible Penrose drain through the chamber with pressure ICP and bypass representing intracranial compartment and extracranial vascular bed. Pd is pressure distal to the clamp. Here Pd, SPP, and ICP are scaled to Pa. Correspondingly, SPP = Pd–ICP and fractional flow reserve (FFR) is Pd/Pa. Experimental data for FFR 77, 50, and 15 are shown. As chamber pressure is increased, Pd rises due to partial obstruction of the collapsible outflow pathway in the Starling chamber. Estimated SPP represents the best fit of the SPP equation. When FFR is 100, SPP is equivalent to the cerebral perfusion pressure CPP. SPP estimation error 0.81 ± 0.57, n = 1226 (scaled to the inflow pressure Pa) is comparable to the manufacturer specified pressure transducer measurement error in the bench system (2% of the reading).

**Figure 3:**
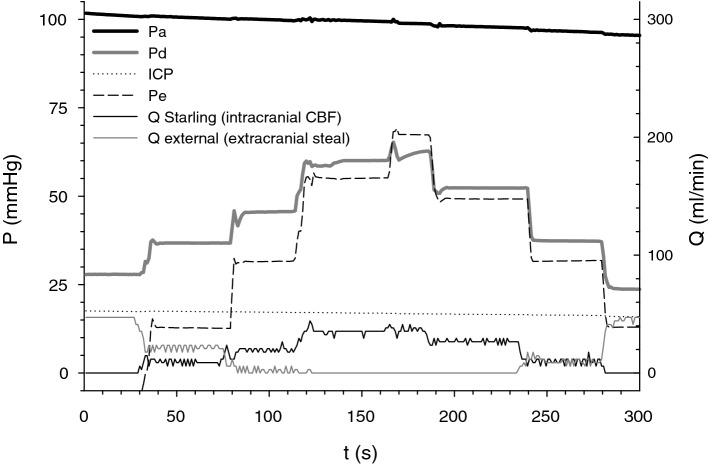
Bench simulation of the intra-extracranial flow diversion with increased intracranial pressure: Fluid from the irrigation set with pressure Pa is flowing through the Starling resistor with pressure ICP and external pathway with outflow pressure Pe. Flow is measured with the rotary flowmeters, where flow Q Starling represents CBF in the intracranial compartment and is zero at the start of measurement. Roller clamp before the bifurcation creates fixed inflow resistance with the pressure Pd, distal to it. As outflow pressure in the external bypass pathway Pe is increased by raising the height of the outflow, flow Q external decreases, and Pd increases, reestablishing Q Starling due to the steal reversal. Maximal flow in the Starling resistor coincides with the cessation of flow in the bypass pathway and with maximization of Pd: Rising Pe beyond this point does not increase Pd and does not increase flow through the Starling resistor. Lowering Pe again, induces flow diversion from the Starling resistor, thus simulating intra-extracranial steal: which depends—as shown—from ICP–Pe (outflow pressure) gradient, and thus is reversible by increased Pe.

To verify the SPP equation, we registered the distribution of free-flowing fluid between the collapsible tube in the pressurized chamber and bypass pathway representing intracranial and extracranial pathways (Figs. 2 and 3).

Intra-extracranial steal (due to the elevated intracranial pressure) and its reversal by extracranial outflow manipulation was simulated in the free-flowing fluid bench (Fig. 3).

### Feasibility of intracranial inflow pressure, and intra-extracranial flow measurement and manipulation in vivo

**Figure 4:**
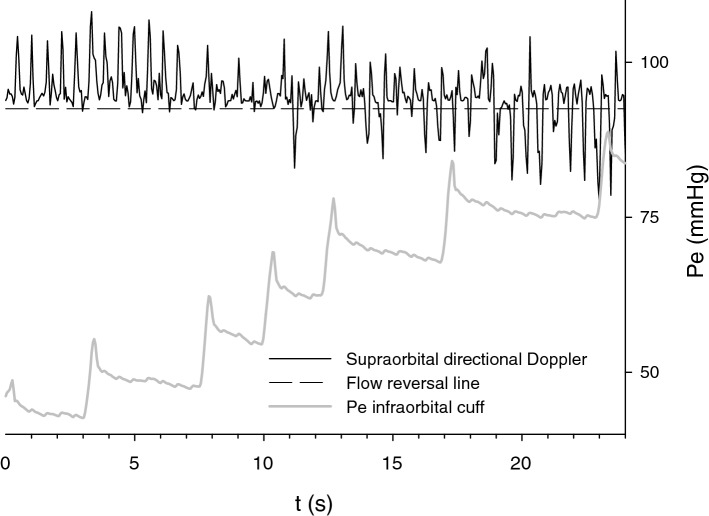
Reversal of the intra-extracranial steal in the supraorbital artery. Directional Doppler examination of the supraorbital artery in a healthy volunteer demonstrates that antegrade (intra-extracranial flow) via this intra-extracranial collateral reverses during step-wise infraorbital cuff inflation: as extracranial outflow pressure, Pe increases. Characteristic oscillations are visible in the infraorbital cuff due to the transmission of the pulse from the facial and temporal arteries (branches of the external carotid).

Intra-extracranial flow through the supraorbital artery was examined in the healthy volunteer and reversed during stepwise inflation of the infraorbital cuff (Fig. 4).

**Figure 5:**
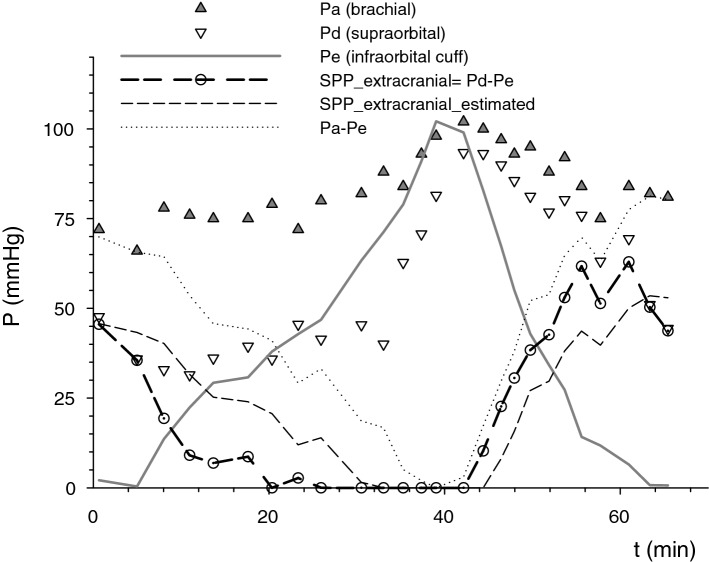
In-vivo measurement of supraorbital pressure with extracranial outflow manipulation: mean systemic pressure Pa was measured using the oscillometric method in the recumbent awake volunteer. Supraorbital pressure Pd (intra-extracranial collateral) was detected by registering maximal photoplethysmographic oscillation during graded compression. At the same time, inflatable infraorbital cuff controlled extracranial outflow pressure Pe. SPP_estimated was calculated using the SPP equation, with fractional flow reserve 0.66, obtained during first measurement, relative extracranial conductance 0.5, and intracranial pressure 0. SPP was assigned the value of 0, when Pe exceeded Pd (complete extracranial outflow obstruction). Extracranial perfusion pressure (Pa–Pe)—the extracranial equivalent of cerebral perfusion pressure—was higher than segmental perfusion pressure (difference 24.6 ± 2.7 mm Hg, P < 0.001), whereas there was no significant difference between SPP_extracranial and it’s estimated value SPP_extracranial_estimated (difference 0.8 ± 2.5, P = 0.82). Inflow pressure Pd increased during infraorbital cuff inflation, R = 0.5, P = 0.01.

Noninvasive measurement of the supraorbital artery (intra-extracranial collateral) pressure Pd demonstrated that intracranial inflow pressure Pd increases with selective extracranial outflow obstruction and measured extracranial SPP approaches zero at the flow reversal point as measured by directional Doppler (Fig. 5).

The difference between systemic and segmental perfusion pressure for the extra-cranial compartment was 24.6 ± 2.7 mm Hg, P < 0.001.

### Simulation of cerebral autoregulation and segmental perfusion pressure with reversible intra-extracranial blood flow diversion

**Figure 6:**
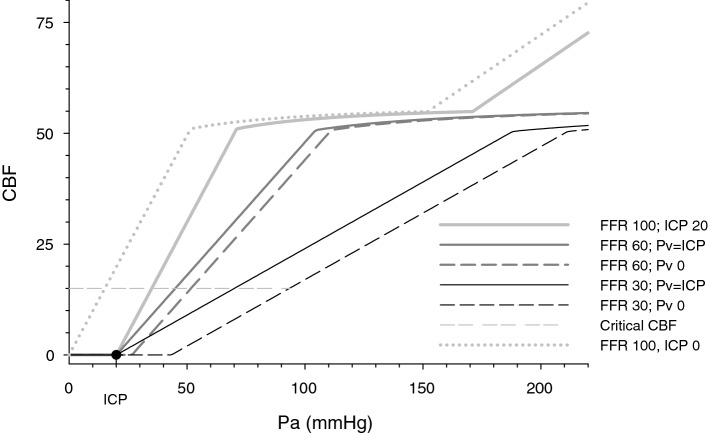
Simulated autoregulation curve (AC) with and without intra-extracranial flow diversion. Simulated autoregulation limits 50–150 mmHg and maximally vasoconstricted to vasodilated intracranial compartment resistance ratio 2.2. Increased intracranial pressure (ICP) and inflow stenosis (FFR) transform AC differently: increased ICP (20 mmHg shown) shifts AC to the right by the value of ICP, while inflow stenosis flattens not autoregulated portion of AC, without altering zero flow pressure. Steal manifests only when intra-extra cranial outflow pressure gradient ICP-Pe coexists with decreased FFR, and it shifts AC (that is already flattened by inflow stenosis) to the right of ICP. These effects are relevant during the low flow states caused by hypotension, severe extracranial stenosis, and high ICP. In this simulation to maintain flow above the critical level with FFR 30 and ICP 20 mmHg, Pa has to exceed 100 mmHg.

Simulation of the cerebral autoregulation with and without intra-extracranial blood flow diversion has demonstrated that decreased FFR shifts autoregulation curve to the right, but does not change zero flow arterial pressure; at the same time, that relationship is shifted to the right by the increased ICP and intra-extracranial steal (Fig. 6). SPP surfaces, that represent zero flow (SPP = 0) and 50% flow (SPP = 0.5*Pa) plotted in the FFR, Pe, and ICP space demonstrate that, because of the extracranial stenosis and extracranial cerebral blood flow diversion, ICP reduces SPP, more so when FFR and Pe are low.

## Discussion

Current neuro-critical management of the injured brain (should it be trauma, stroke, or a space-occupying lesion) is driven by the equation of cerebral perfusion pressure (CPP)^4–8^. Although this equation accounts for the outflow pressure in the intracranial compartment (ICP), it does not address systemic to an intracranial arterial pressure gradient (Pa-Pd) due to extracranial vascular stenosis^1,9^. Because autoregulating vessels are distal to the circle of Willis, they are exposed to the Pd—inflow pressure of the intracranial compartment, not the systemic pressure Pa. Hence, segmental cerebral perfusion pressure (SPP = Pd–ICP) is more applicable to cerebral microcirculation, than is the systemic perfusion pressure (CPP = Pa–ICP). This is the reason why Pd, rather than Pa, is used to determine the need for shunting during carotid endarterectomy (it is measured directly as carotid stump pressure), after carotid cross-clamp^10^.

Pd can be expressed as load-independent index- fractional flow reserve FFR = Pd/Pa in the completely vasodilated state, which determines the hemodynamic significance of inflow stenosis^11,12^. And it was recently applied to the cerebral vasculature^1,13^. Direct cerebral FFR measurement in vivo turned out to be feasible and reproducible^9^. It can be measured noninvasively as a ratio of ophthalmic to brachial artery pressure (ophthalmic pressure index) using ophthalmomanometry-Doppler^14^, or it can be estimated by measuring the pressure in the supraorbital artery (intra-extracranial collateral)^15,16^. Alternatively, FFR can be estimated from the CT angiograms, by applying patient-specific models of fluid dynamics^17^.

The concept of SPP can be expressed by one equation—the equation of intracranial compartment perfusion pressure—which accounts not only for arterial pressure and ICP, but also for the pressure drop due to extracranial stenosis and intra-extracranial steal. Thus, segmental cerebral perfusion pressure could substitute CPP as a more precise individualized therapeutic target in neurocritical care of traumatic brain injury, stroke, in hypertensive crisis, and anesthesia in sitting position^5,18–20^.

As it can be seen from the SPP equation: if ICP is minimized medically and/or by the surgical intervention, but SPP is still below the autoregulatory limit despite blood pressure augmentation, intervention to address extracranial stenosis (endarterectomy or stenting) may be necessary. It will decrease FFR and will restore autoregulation^21^ (autoregulation shift to the left comparing FFR 30 to 60 in Fig. 6). Variation in SPP could explain why a subset of patients have decreased intracranial blood flow after decompressive craniectomy^22^. In the presence of intra-extracranial flow diversion, lowering ICP increases SPP more than equivalent CPP increase, without decreased ICP. This explains the observation that CPP could be reduced from 60 to 35 mmHg if decompressive craniectomy controls elevated ICP, without increasing mortality in patients with the severe traumatic brain injury^4^.

**Figure 7:**
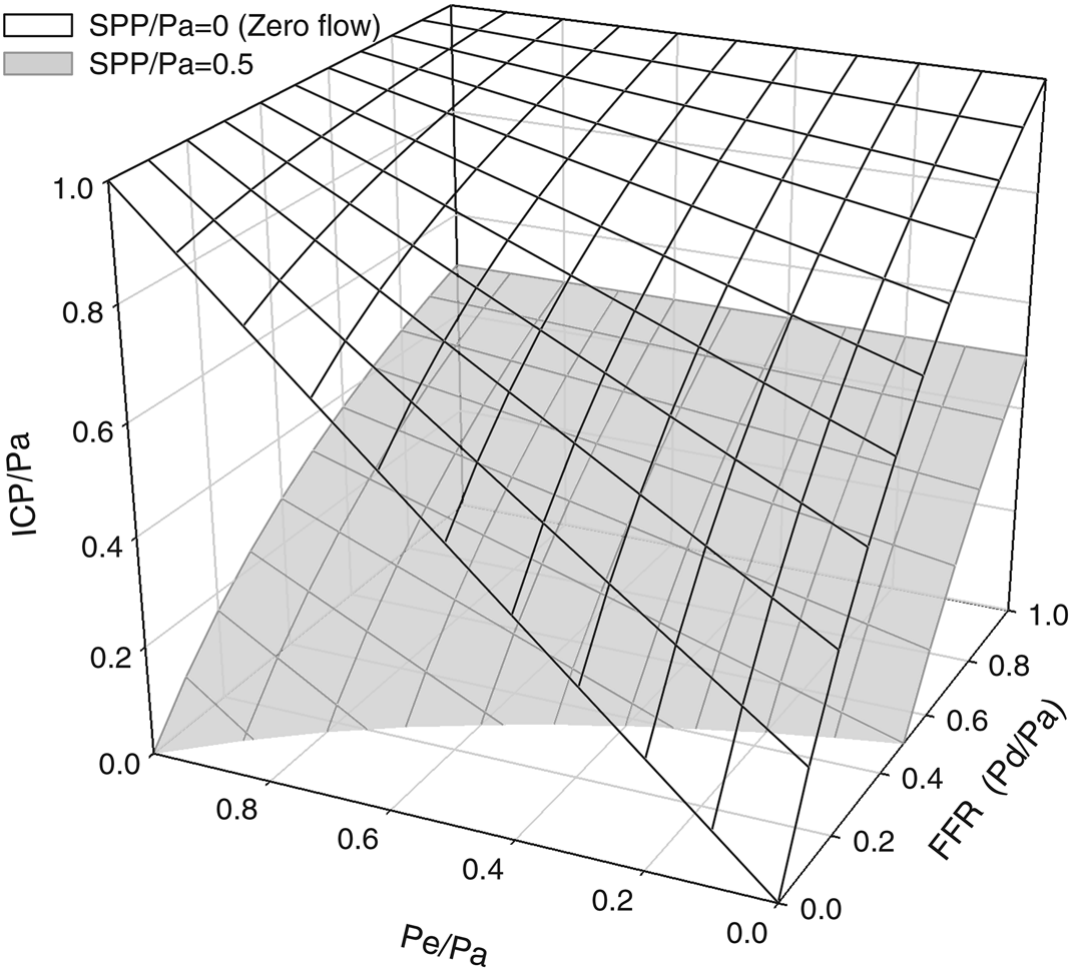
Surfaces indicating zero and 50% (of arterial pressure Pa) intracranial segmental perfusion pressure—the difference between intracranial inflow pressure Pd and intracranial pressure ICP (SPP = Pd–ICP); SPP surfaces were calculated from the intra-extracranial blood flow model expressing ICP from the fixed SPP value. Surfaces reflect all possible combinations of FFR, ICP, and Pe leading to SPP being either 0 or 50% of Pa, where FFR = Pd/Pa is fractional flow reserve. Extracranial outflow pressure (Pe), ICP, and SPP—are all scaled to the arterial pressure Pa. Intra-extracranial conductances were assumed to be equal (relative extracranial conductance Ge = 0.5). Autoregulation was presumed absent. Zero SPP surface indicates the absence of intracranial flow. When FFR or Pe is 1, ICP has to reach arterial pressure to make SPP zero, thus arresting blood flow. When FFR is low, intracranial blood flow stops with lower ICP due to intra-extracranial steal, which can be reversed by increasing extracranial outflow pressure. The specific location of the zero flow condition suggests most effective resuscitative measures to increase SPP to 50% of Pa: with ICP close to arterial pressure, the only way to increase SPP is to lower ICP, however, if zero flow conditions exist at relatively low ICP, addressing extracranial stenosis is more important, while at the same time extracranial outflow manipulation could be used for temporary reversal of the intra-extracranial steal.

SPP equation can explain why blood flow studies to support brain death diagnosis have false positives or false negatives^23^. Brain death in the terminal stages of traumatic brain injury is associated with increased intracranial pressure that confounds evaluation of the cerebral blood flow cessation. Intracranial blood flow ceases when segmental perfusion pressure decreases to zero, although this can be mitigated by increasing blood pressure or by decreasing ICP (Fig. 7, zero flow SPP surface). False-positive results of the angiography in hypotensive patients and false-negative results of scintigraphy or transcranial Doppler after decompressive craniectomy—that were referenced by Gastala et all^23^—can be explained by the SPP equation which accounts for intra-extracranial flow redistribution.

A similar blood flow redistribution phenomenon at the interhemispheric level was discovered by Bhaskar et all. who demonstrated delayed late-venous phase cortical vein filling in a subgroup of acute ischemic stroke patients, which was associated with large artery atherosclerosis, low collateral grade, and low-flow state^24^.

To evaluate regional blood flow phenomena, the SPP equation for the intracranial compartment needs to be resolved as a regional segmental cerebral perfusion pressure map similar to the FFR map^17^: that could account not only for the intra-extracranial but also for the extracranial, interhemispheric, and intrahemispheric venous collaterals^25,26^, thus incorporating timed regional venous filling maps^24,26^.

Such mapping could explain recently discovered delayed venous filling^24^ and extracranial blood flow diversion to the epidural and vertebral venous plexus with increased intracranial pressure^27^.

For the simulation of the interhemispheric flow distribution, model of Piechnik et al. could be used^28^, substituting Pa with Pd from the SPP Eq. (1). Piechnik et all investigated cerebral blood flow response to the lowering of blood pressure and hypoventilation and found that in the absence of the extracranial stenosis, the interhemispheric pressure gradient across the anterior communicating artery did not exceed 1.2 mmHg. Their simulation supports the use of the circle of Willis as a surrogate for cerebral inflow. The authors did not demonstrate significant interhemispheric steal when extracranial stenosis was absent and there was no ICP gradient between hemispheres.

Both bench and volunteer tests demonstrated that intra-extracranial steal due to the ICP-Pe gradient is reversible when extracranial outflow pressure Pe is selectively augmented to exceed ICP (Figs. 4 and 5). This makes possible augmentation of intracranial perfusion by manipulating the extracranial outflow. We demonstrated the reversibility of the intra-extracranial blood flow diversion via supraorbital artery inflating infraorbital cuff up to the level of mean arterial pressure. Safety of long-term use of such a cuff is not established. However it is reasonable to assume that extrapolating data from the CPAP therapy, pressures up to 20–30 cm H20 may be used for the longer term with the properly designed infraorbital cuff^29^. The ophthalmic artery is one of several intra-extracranial anastomotic pathways^2^, hence, infraorbital cuff does not reverse intra-extracranial flow diversion via ascending pharyngeal or vertebral arteries^30^. However vertebral blood flow could be augmented by the inflation of the brachial cuff^31^ and lower body counterpulsation^32^. Hence, the reversal of the flow, that was demonstrated in the intra-extracranial collaterals (supraorbital and in the vertebral arteries) by manipulating extracranial compartment outflow pressure, has far-reaching therapeutic implications. It could be used for the cerebral blood flow augmentation and delivery of therapeutics to both, anterior and posterior cerebral circulation.

Thevenin’s equivalent representation of the inflow circuit presumes linear pressure/flow characteristics and can not be used to model nonlinear wave phenomena or to account for non-Newtonian viscosity. Another limitation is the non-linearity of the Starling resistor concept itself: Starling resistor is the formula for the idealized blood flow in collapsible vessels with low/negative transmural pressures which presumes that effective outflow pressure is determined by the external pressure.

Given these limitations, we analyzed the non-linear Starling resistor that is exposed to increased external pressure using an electrical circuit model with nonlinear elements^12^. The analysis has demonstrated that inflow resistance dependent steal is present in the context of the pulsatile blood flow and nonlinear vessel compliance. Moreover, it is not qualitatively different from the Thevenin approximation of intra-extracranial blood flow distribution. Likewise, a physical bench study with an irrigation set also demonstrated a close approximation of measured SPP by the Thevenin estimate (Fig. 2). Thus, given West zone 2 conditions, when Pd > ICP ≥ Pe (intra-extracranial steal), or Pd > Pe ≥ ICP (extra-intracranial steal)^33^, Thevenin approximation of extracranial inflow is applicable to the linear portion of the Starling resistor.

For the application of the SPP equation in vivo, FFR and Ge have to be estimated. Extracranial conductance fraction Ge was fitted from the empiric data in bench and in-vivo study. It can be estimated from CT angiography, MRA, or carotid duplex (by quantifying intracranial and extracranial flow separately). SPP formula presumes fixed outflow resistance, and is valid below the lower limit of the autoregulation, when cerebral vasculature is maximally vasodilated. If autoregulation is intact (incomplete cerebral vasodilation), Ge may be overestimated. Intact autoregulation contributes to the extracranial SPP estimation error (2.3 ± 15.1 mmHg, n = 26, measured noninvasively with the photoplethysmographic method, Fig. 5).

Strauss et al. measured the impact of the extracranial stenosis (FFR) noninvasively as an ophthalmic artery index (ratio of opthalomomanometric-Doppler ophthalmic to brachial pressure) and found it to be 0.68 ± 0.04 in patients without carotid stenosis, 0.54 ± 0.08 in patients with carotid stenosis, and 0.46 ± 0.08 in patients with carotid artery occlusion (p < 0.001). In the longitudinal study, they saw small day to day variation of the said FFR measurement (SD 0.04). In 37 patients with occlusive carotid artery disease, Doppler ophthalmic pressure index increased from 0.59 ± 0.07 to 0.71 ± 0.05 (p < 0.001) after carotid endarterectomy^34^. The sensitivity of the supraorbital pressure measurement to the degree of the carotid stenosis was demonstrated using direct^16^, photoplethysmographic^15^ and Doppler measurements of the supraorbital artery pressures^35^.

And finally: in the absence of stenosis, the SPP formula collapses into the standard CPP equation (SPP≈CPP when FFR≈1). The sign of “approximate” indicates the fact that even vessels without stenosis have resistance. Within these constraints, the SPP equation can be used for the conceptualization, measurement, and management of intracranial compartment perfusion pressure and intra-extracranial blood flow distribution.

From all the above follows possible clinical applications of the SPP equation framework for the diagnosis and management of cerebral blood flow. Firstly, cerebral inflow pressure Pd can be estimated for the intra-extracranial collaterals (like supraorbital artery). Secondly, the patient-specific SPP equation can be used to establish the individual cerebral perfusion target. Thirdly, hemodynamic effects of ICP reduction, arterial pressure augmentation, and carotid endarterectomy/stenting can be interpreted based on their effects on SPP^36–38^. And finally, extracranial outflow manipulation can be used for temporary cerebral blood flow augmentation and to enhance the delivery of various therapeutics into the intracranial compartment: such as thrombolytics, cerebral protection agents (including cooling), antibiotics, chemotherapeutics, anesthetics, and vasoactive substances. Of course, further experimental and clinical studies will be necessary to verify the SPP equation applicability in patients with various degrees of extracranial stenosis and increased intracranial pressure, as well as the feasibility of intra-extracranial steal reversal to augment cerebral blood flow and to deliver therapeutics in the clinical setting.

## Methods

Although cerebral blood supply is redundant with two pairs of inflow arteries and multiple extra-intracranial collaterals and venous outflow pathways^2^, by applying Thevenin’s theorem to intra-extracranial circulation^39,40^, all intra-extracranial inflow pathways can be substituted by one equivalent pressure source (Pa) and one inflow resistance (Ri); whereas pressure distal to that inflow resistance is average intracranial inflow pressure at the circle of Willis Pd. Using this simplification we studied flow distribution between intracranial and extracranial pathways with outflow pressures being ICP and Pe (Fig. 1).

We applied our earlier cerebral venous steal concept—the concept that describes blood flow distribution between cerebral regions with different tissue pressures^3^—to the distribution of flow between the intracranial and extracranial compartments with corresponding outflow pressures of ICP and Pe; whereas relative pathway resistances were expressed in terms of the FFR for Thevenin equivalent of intra-extracranial inflow and of relative extracranial conductance Ge (Fig. 1 and Appendix).

The segmental perfusion pressure equation for the intracranial compartment is the modification of a widely known cerebral perfusion pressure (CPP) equation: CPP = Pa–ICP. Instead of applying perfusion pressure equation globally, it accounts for the pressure gradient Pa–Pd due to the extracranial stenosis and applies perfusion pressure equation separately to the regions with different outflow pressure: for intracranial compartment SPP = Pd–ICP, and for extracranial compartment it is Pd–Pe, where Pd is common inflow pressure for intracranial and extracranial compartments and Pe is extracranial outflow pressure. SPP formula adjusts intracranial compartment perfusion pressure for the degree of extracranial stenosis (fixed) and for the intra-extracranial steal (variable, depending on the ICP–Pe gradient). It expresses intracranial perfusion pressure in the terms of FFR = Pd/Pa measured at the circle of Willis, cerebral perfusion pressure, and the degree of steal (1, Fig. 7).

Following is the discussion of each constituent term in the SPP equation separately.

On the left side of the SPP equation is the singular variable—segmental perfusion pressure (SPP): it is the residual, and the sole, force that drives perfusion in the intracranial compartment.

Then, the first term of the right side of the equation (FFR•CPP) indicates that SPP is an extension of widely known CPP: the difference being, that SPP accounts for the pressure drop across the inflow and the collateral pathways combined. And while it is customary to think that primary inflow and collaterals are separate entities, SPP is “blind” in this regard, reflecting the composite measure of both, lumped together under Thevenin’s equivalent of “inflow segment,” which has its combined resistance. The higher is inflow resistance, and/or the higher is blood flow (including extracranial pathway), the higher is the difference between Pa and Pd, and corresponding SPP becomes lower (Figs. 2, 7). All of that is in stark contrast with the classic understanding of CPP which does not change when the resistance of inflow changes. Carotid stump pressure therefore rather than systemic pressure is used to assess the hemodynamic significance of carotid cross-clamp^10,41^. SPP and CPP are equivalent only when extracranial stenosis is absent (FFR≈1): an approximation of the situation in the pediatric population without intrinsic carotid artery disease (Figs. 2, 7 FFR 100).

The second term in the SPP equation represents Pd decrease secondary to the intra-extracranial steal: this term approaches zero when either FFR is 1 or ICP-Pe is 0, thus making the product of their multiplication zero (instances when steal does not exist).

The mathematical model of intra-extracranial blood flow diversion was verified in the physical bench, by measuring pressure and flow distribution in the irrigation set with the inflow pressure Pa that corresponds to the height of the set. Adjustable roller clamp represented inflow resistance Ri with pressure Pd distal to it. Flow through the Starling resistor (Penrose drain suspended in the chamber with pressure “ICP”) represented the intracranial compartment, while the extracranial flow was represented by the parallel outflow line with variable outflow height (Pe) and was measured by the flowmeters. Pressures Pa, Pd, ICP, and Pe were acquired using BD, Franklin Lakes, NJ pressure transducers, and 4 channel 24 bit analog to digital converter; rotary flow meters output was recorded by two edge counters at 2 Hz. SPP represented the difference between Pd and chamber pressure ICP, and the FFR- a ratio of Pd to Pa. SPP was measured during the change in chamber pressure, with FFR 77, 50, and 15. Flow distribution between extra-intracranial compartments was simulated changing Pe, while chamber pressure that represented ICP was held constant.

For the in-vivo feasibility study, Albert Einstein College of Medicine IRB approval 2019-11008/059896 was obtained; informed consent was signed by the consenting investigator. All procedures performed in the human participant were in accordance with the ethical standards of the institutional research committee and with the 1964 Helsinki Declaration and its later amendments or comparable ethical standards.

Forehead skin capillary pressure was measured using the photoplethysmographic method^15^- Parks Medical Electronics, Aloha, OR photoplethysmographic probe with the interpositioned pediatric blood pressure cuff was fitted under the rigid headband overlying the supraorbital region. Another circumferential infraorbital cuff fitted over the nasal mask to preserve nasopharyngeal air passage controlled extracranial venous outflow mimicking graded compression by the endotracheal tube holder. Mean brachial blood pressure was measured using ambulatory oscillometric blood pressure monitor ABPM50 (Contec Medical Systems, Hebei, China). The photoplethysmographic output from the vascular flowlab (Parks Medical Electronics, Aloha, OR) and calibrated pressure signals from the cuffs were digitized using the data acquisition system for the bench test.

After inflation of the overlying cuff, supraorbital plethysmographic oscillation disappeared, then reappeared during gradual deflation; when it reached maximal amplitude, it was assumed that cuff pressure is equal to the mean supraorbital pressure Pd^15^. Measurements were repeated during stepwise changes in the infraorbital cuff pressure Pe partially occluding extracranial outflow to induce extra-intracranial rather than intra-extracranial steal which would require invasive measurements and could not be completed throughout the full range of ICP without compromising cerebral blood flow. Intra-extracranial flow through the supraorbital artery was recorded using 8 MHz directional Doppler (Parks Medical Electronics, Aloha, OR) during stepwise changes in the infraorbital cuff pressure^42^.

### Compliance with ethical standards

All measurements in a human participant were performed in accordance with the ethical standards of the institutional research committee and with the 1964 Helsinki Declaration and its later amendments.

### Ethical approval

All measurements in a human participant were performed with Albert Einstein College of Medicine IRB approval 2019-11008/059896 and informed consent of the subject.

## Conclusion

Separation of the blood flow into intracranial and extracranial pathways allows quantification of perfusion pressure for the intracranial compartment that accounts for the inflow stenosis and intra-extracranial flow diversion. Inflow pressure for the intracranial compartment can be measured in the extracranial collaterals (supraorbital artery). Reversibility of the intracranial-extracranial steal—that is predicted by the SPP equation and demonstrated in the bench and in-vivo measurement—presents an opportunity to augment cerebral blood flow by selective manipulation of the extracranial outflow.

## Supplementary Information

Supplementary Information.
