# Supplementary material for: Cerebral venous steal equation for intracranial segmental perfusion pressure predicts and quantifies reversible intracranial to extracranial flow diversion
Source: Sci Rep. 2021 Apr 8;11:7711. doi: 10.1038/s41598-021-85931-x (PMC8032738; doi:10.1038/s41598-021-85931-x)
Supplement: Supplementary file 1 — Supplementary Information. [file 41598_2021_85931_MOESM1_ESM.docx]

### **Supplementary material:**

### **Equations of the segmental perfusion pressure (SPP)**

**Article title:**

**Cerebral venous steal equation for intracranial segmental perfusion pressure predicts and quantifies reversible intracranial to extracranial flow diversion**

**Authors**

Mindaugas Pranevicius^1^, MD; Henrikas Pranevicius^2^, Dr. Hab., Osvaldas Pranevicius^3^, MD, PhD

^1^ Associate professor of Anesthesiology, Albert Einstein College of Medicine, Bronx, NY, pranevicius@gmail.com

^2^ Professor, Simulation of Complex Systems Laboratory, Kaunas University of Technology, Kaunas, Lithuania, Henrikas.pranevicius@ktu.lt

^3^ Attending Anesthesiologist, Department of Anesthesiology, New York-Presbyterian/Queens, New York, NY, [opranevicius@aol.com](mailto:opranevicius@aol.com)

### **Expression of segmental resistances in terms of FFR and extracranial conductance**

Total outflow resistance for the circuit in fig. 1 with inflow, intracranial, and extracranial resistances **Ri**, **Ro,** and **Re** is:

**R_OUTFLOW_=(Ro·Re)/(Ro+Re) . (1)**

Total circuit resistance is:

**R_TOTAL_=Ri+R_OUTFLOW_ . (2)**

If all resistances are divided by **R_TOTAL_**, then scaled resistances **R^S^i, R^S^o and R^S^e** all can be expressed from the fractional flow reserve (FFR) and the ratio of external conductance to total (intra-extra cranial conductance) **Ge.**

When steal is absent ( Pe=ICP=0), according to the Ohms law for resistances in series, FFR, as the ratio of pressure gradients Pd and Pa (after factoring out flow Q) simplifies to the ratio of **R_OUTFLOW_** to **R_TOTAL_:**

**FFR=** Pd/Pa=(Q**·R_OUTFLOW_**)/(Q**·R_TOTAL_**)=**R_OUTFLOW_ /R_TOTAL_=R^S^_OUTFLOW_. (3)**

**R^S^i=1-FFR. (4)**

**R^S^e=FFR/ Ge. (5)**

**R^S^o=FFR/(1- Ge). (6)**

The expression for post-stenotic pressure Pd was adapted from Pranevicius^1^; namely for Starling resistor, Zone 2 conditions (Pd>ICP and Pd>Pe), where outflow pressure in the intracranial compartment is ICP, and outflow pressure in the extracranial steal pathway is Pe:

**Pd=(Pa/ R^S^i +ICP/ R^S^o +Pe/ R^S^o)/(1/ R^S^i +1/ R^S^o +1/ R^S^o).**

Substituting resistances with their expressions, using FFR and **Ge**, the following expression for post-stenotic pressure is obtained:

**Pd=FFR·Pa+(1- Ge) · (1-FFR) ·ICP+Pe· Ge · (1-FFR). (7)**

Then segmental perfusion pressure (SPP) is:

**SPP=Pd-ICP=FFR· (Pa-ICP)- Ge · (1-FFR) · (ICP-Pe). (8)**
ICP can be expressed from the SPP equation (8):

**ICP=( Ge · (1-FFR) · Pe + FFR·Pa - SPP) / (FFR+ Ge · (1-FFR)) (9)**

1. Pranevicius, M. & Pranevicius, O. Cerebral venous steal: blood flow diversion with increased tissue pressure. *Neurosurgery* **51**, 1267-73; discussion 1273-4 (2002).
